# Multiple Pulmonary Cavernous Hemangioma: A Case Report

**DOI:** 10.70352/scrj.cr.25-0537

**Published:** 2025-11-22

**Authors:** Shinogu Takashima, Hiroshi Nanjo, Tsubasa Matsuo, Shoji Kuriyama, Hidenobu Iwai, Haruka Suzuki, Mirai Kobayashi, Tatsuki Fujibayashi, Sumire Shibano, Yoshihiro Minamiya, Kazuhiro Imai

**Affiliations:** 1 Department of Thoracic Surgery, Akita University Graduate School of Medicine, Akita, Akita, Japan; 2 Department of Pathology, Akita University Hospital, Akita, Akita, Japan

**Keywords:** cavernous hemangioma, PET-CT, pulmonary hemangioma, case reports

## Abstract

**INTRODUCTION:**

Pulmonary cavernous hemangioma (PCH) is extremely rare and, due to the lack of specific radiological characteristics, is often misdiagnosed as other pulmonary diseases, including metastatic tumors. Here, we report a case of multiple PCH lesions with concomitant hepatic cavernous hemangioma (HCH), emphasizing the diagnostic implications of imaging findings.

**CASE PRESENTATION:**

A 57-year-old man presented with fever. CT revealed numerous well-circumscribed pulmonary nodules and multiple low-density hepatic lesions. Dynamic contrast-enhanced CT demonstrated mild, nonspecific enhancement of the pulmonary nodules, whereas the hepatic lesions showed enhancement from the center to the periphery. Serum tumor markers were within normal limits, and PET-CT revealed no abnormal fluorodeoxyglucose uptake in any of the lesions. Although the hepatic lesions appeared suggestive of hemangioma on imaging, the possibility of malignancy, such as hepatic angiosarcoma and its pulmonary metastases, could not be completely ruled out. Therefore, both hepatic and pulmonary biopsies were performed, and histopathological examination confirmed cavernous hemangioma in both organs.

**CONCLUSIONS:**

PCH is a rare benign tumor lacking distinctive imaging characteristics; therefore, differentiation from metastatic pulmonary tumors is often challenging and represents a key diagnostic issue. Although definitive diagnosis currently relies on surgical biopsy, combining suggestive imaging findings—such as the presence of microcalcifications within nodules and the absence of fluorodeoxyglucose uptake on PET-CT—may help avoid unnecessary invasive procedures.

## Abbreviations

HCH
: hepatic cavernous hemangioma
HHT
: hereditary hemorrhagic telangiectasia
PCH
: pulmonary cavernous hemangioma

## INTRODUCTION

Pulmonary cavernous hemangioma (PCH) is a rare benign tumor of the lung,^1)^ and most reports in the literature are single-case descriptions.^2–12)^ According to a recent review, only about 40–50 cases have been reported in total, and cases with multiple lesions account for only one-third of them.^13)^ Concomitant occurrence of PCH with hepatic cavernous hemangioma (HCH) is even rarer, with only a few such cases reported.^2,4,12)^

PCH lacks distinctive radiological features, making differentiation from metastatic pulmonary tumors difficult, particularly in patients with a history of malignancy or in cases involving space-occupying lesions in other organs. Here, we report the case of a 57-year-old man with multiple PCH lesions associated with HCH and discuss the diagnostic considerations and clinical significance.

## CASE PRESENTATION

**Fig. 1:**
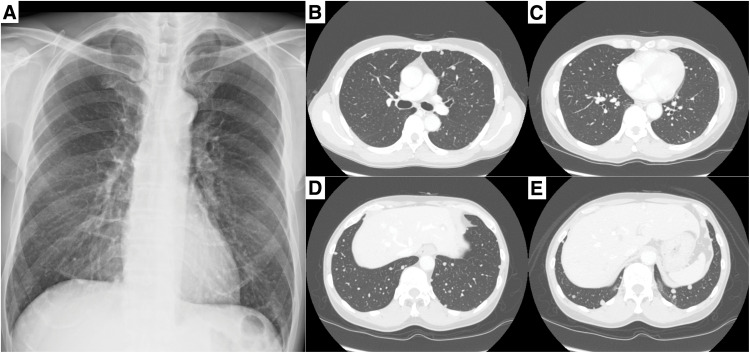
Chest X-ray showing multiple small nodules predominantly in the middle and lower lung fields of both lungs (**A**). Plain CT revealed multiple well-circumscribed nodules up to 1.0 cm in diameter in the bilateral lungs (**B**–**E**).

**Fig. 2:**
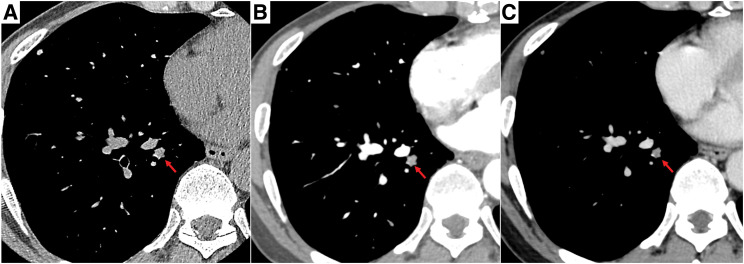
Plain CT (**A**), early-phase contrast-enhanced CT (**B**), and delayed-phase contrast-enhanced CT (**C**) are shown. The pulmonary nodules exhibited nonspecific mild enhancement, and peripheral microcalcifications were observed in approximately one-third of the nodules (representative lesions are indicated by red arrows).

**Fig. 3:**
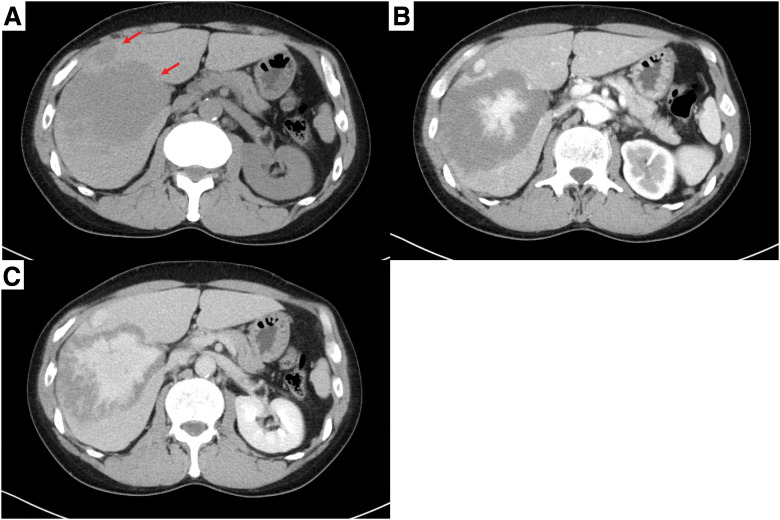
Abdominal CT images. Plain CT revealed multiple homogeneous low-density lesions (red arrows) (**A**). Dynamic contrast-enhanced CT demonstrated gradual enhancement extending from the center toward the periphery in the arterial phase (**B**), with persistent enhancement in the equilibrium phase (**C**).

A 57-year-old man presented to a nearby hospital with fever. Chest X-ray revealed multiple nodules predominantly in the middle and lower lung fields of both lungs (**Fig. 1A**). Chest CT demonstrated multiple well-circumscribed pulmonary nodules (**Fig. 1B**–**1E**), and contrast-enhanced CT showed mild, nonspecific enhancement of these nodules. In approximately one-third of all nodules, peripheral microcalcifications were also observed (**Fig. 2A**–**2C**). Abdominal CT revealed multiple low-density lesions in both hepatic lobes (**Fig. 3A**). On dynamic contrast-enhanced CT, the hepatic lesions showed enhancement extending from the center toward the periphery in the arterial phase, with progressive and persistent enhancement in the equilibrium phase (**Fig. 3B** and **3C**). These imaging findings suggested benign hepatic hemangiomas, and histological examination of a liver biopsy performed at the referring hospital was also consistent with HCH. However, because the hepatic lesions were multiple and the histological diagnosis was based on a biopsy from a single lesion, the possibility of pathological heterogeneity could not be completely excluded. In particular, given the coexistence of multiple pulmonary nodules, metastatic disease from a potentially malignant hepatic lesion could not be definitively ruled out. Therefore, the patient was referred to our hospital for further evaluation of the pulmonary nodules.

**Fig. 4:**
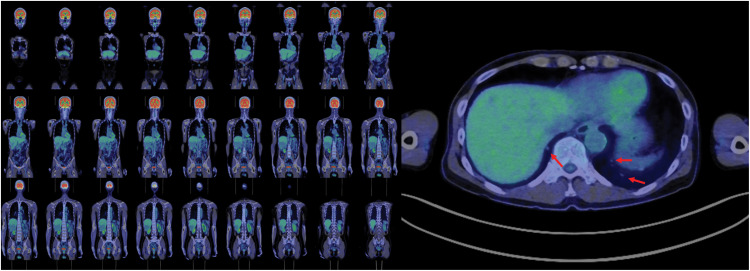
PET-CT images. Neither the pulmonary nodules nor the liver tumors showed abnormal fluorodeoxyglucose uptake (lung nodules are indicated by red arrows).

PET-CT performed 3 months after the initial CT revealed no abnormal fluorodeoxyglucose uptake in either the pulmonary or hepatic lesions, and no interval increase in lesion size was observed (**Fig. 4**). The patient had no comorbidities, no family history of PCH, and physical examination revealed no remarkable findings, including skin manifestations. Serum tumor markers were within normal limits, and serum biochemical, pulmonary, and cardiac function tests showed no abnormalities.

**Fig. 5:**
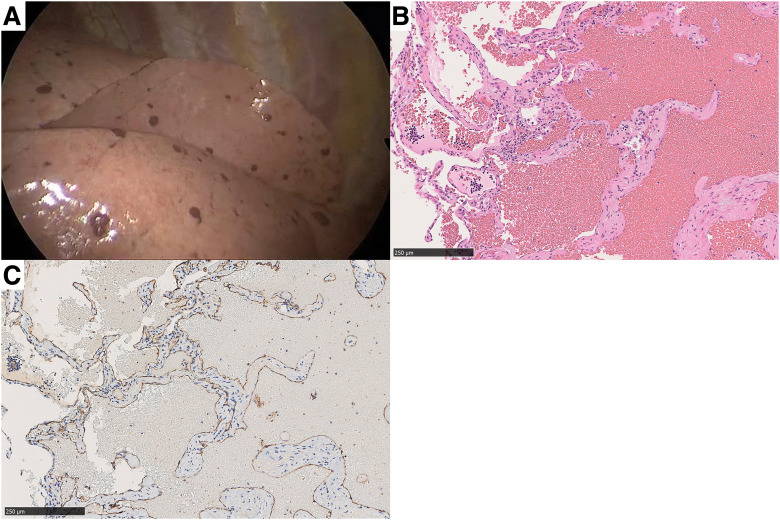
Thoracoscopic views revealed multiple dark-red nodules on the surface of the visceral pleura (**A**). Histopathological examination (hematoxylin/eosin stain) revealed aggregates of vein-like structures with fibrous septa and lumens filled with red blood cells (**B**). Endothelial cells were positive for CD31 (**C**).

Uniportal video-assisted thoracic surgical biopsy was performed on 1 of the right middle lobe nodules. Macroscopically, multiple dark-red pulmonary nodules up to 1.0 cm in diameter were observed on the visceral pleural surface (**Fig. 5A**). Microscopically, the lesion was diagnosed as PCH, characterized by a cavernous hemangioma composed of vein-like structures with fibrous septa and lumens filled with red blood cells (**Fig. 5B**). Immunohistochemical staining demonstrated CD31 positivity in the endothelial cells of the cavernous structures, confirming the vascular origin (**Fig. 5C**).

The patient was discharged without complications on the 5th POD and remains under follow-up. Postoperative brain MRI showed no intracranial lesions, including hemangiomas. Chest CT obtained 6 months after the pulmonary biopsy demonstrated no interval change in the number or size of PCH. Hepatic MRI performed at the same time revealed multiple T2-weighted hyperintense lesions within the liver, consistent with HCH, with no change in size compared with preoperative imaging. The patient has since been followed by pulmonary and hepatology specialists.

## DISCUSSION

Cavernous hemangiomas affect both sexes equally and can occur anywhere in the skin or subcutaneous tissue; although the liver is the most frequently involved internal organ, pulmonary involvement is extremely rare.^1–3)^ Most patients with PCH remain asymptomatic, but some may present with chest pain,^4)^ dyspnea,^5)^ or, in rare cases, severe hemoptysis.^6)^ The etiology and pathogenesis of PCH remain unclear. Some reports have suggested that PCH may represent a subtype of pulmonary arteriovenous malformation associated with Osler–Weber–Rendu syndrome or hereditary hemorrhagic telangiectasia (HHT).^7–9)^ However, no consistent association has been established. In our case, the patient was asymptomatic, and multiple pulmonary nodules were incidentally detected during a workup for fever. The coexistence of hepatic tumors prevented complete exclusion of metastasis, ultimately resulting in the need for 2 invasive biopsies. This highlights the challenge of diagnosing cavernous hemangioma solely based on imaging.

HCH can generally be diagnosed based on its characteristic imaging features. According to the Japanese guidelines,^14)^ dynamic contrast-enhanced CT typically shows peripheral nodular enhancement in the arterial phase, followed by progressive centripetal fill-in and persistent enhancement in the equilibrium phase. In addition, calcifications are observed in approximately 10%–20% of cases.^15)^ These calcifications are thought to result from thrombus formation caused by blood flow stagnation and local circulatory disturbance within the vascular spaces, followed by organization and calcification over time, leading to the development of phleboliths.^16)^ For hemangiomas showing these typical imaging features, biopsy is generally discouraged because of the risk of bleeding and the high diagnostic accuracy of imaging.^14)^ In the present case, however, the hepatic lesions were suggestive of a benign vascular tumor but demonstrated a slightly atypical enhancement pattern—showing enhancement extending from the center toward the periphery—and no calcification was identified. Because of these atypical findings and the coexistence of multiple pulmonary nodules, malignancy could not be completely excluded, and a liver biopsy was therefore performed.

PCH, particularly when multiple, typically presents as small nodules. This results in slow intralesional blood flow, making it difficult to exhibit the characteristic enhancement pattern commonly observed in HCH,^17)^ and therefore, differentiation from metastatic pulmonary tumors can be challenging. However, such sluggish circulation may promote thrombosis and subsequent calcification, potentially leading to a higher frequency of calcification in PCH than in HCH. In the present case, peripheral microcalcifications were observed in approximately one-third of the nodules. Previous reports on PCH have shown variable descriptions regarding the presence of calcification—some clearly describing it,^18,19)^ while others either made no mention of it or explicitly noted its absence^2)^—indicating that this finding remains controversial. Nevertheless, careful attention to this imaging feature may still provide a useful diagnostic clue. PET-CT has also been suggested as a useful modality for differentiating PCH from other pulmonary diseases. In our case, the nodules were small, which may account for the lack of fluorodeoxyglucose uptake. To date, however, abnormal fluorodeoxyglucose uptake on PET-CT has not been reported in PCH.^2–4,7)^ Taken together with the presence of microcalcifications, these findings may aid in the noninvasive differentiation of PCH from metastatic or other malignant pulmonary lesions.

In cases with multiple PCH lesions, long-term observation is generally considered appropriate as long as the patient remains asymptomatic and the nodules remain stable in size.^20)^ Although no standardized follow-up interval has been established, several reports recommend periodic clinical and radiological assessments every 6 to 12 months to confirm lesion stability.^18,19)^ If symptoms develop, surgical resection of the responsible lesion or alternative therapeutic approaches may be required. For instance, successful treatment with interferon alfa-2a has been reported in a 7-year-old boy with respiratory failure and hemoptysis.^21)^ In addition, although evidence remains limited, mTOR inhibitors such as sirolimus have shown efficacy in vascular malformations and may have potential applicability.^22,23)^

## CONCLUSIONS

PCH is a rare benign tumor lacking distinctive imaging characteristics. Currently, definitive diagnosis relies on surgical biopsy. Nevertheless, the presence of microcalcifications within nodules and the absence of fluorodeoxyglucose uptake on PET-CT may serve as useful diagnostic clues, potentially helping to avoid unnecessary invasive procedures.

## DECLARATIONS

### Availability of data and materials

The data will be made available from the corresponding author on reasonable request.
